# Code-Switching Strategies: Prosody and Syntax

**DOI:** 10.3389/fpsyg.2020.02130

**Published:** 2020-09-23

**Authors:** Rena Torres Cacoullos

**Affiliations:** Department of Spanish, Italian and Portuguese, The Pennsylvania State University, University Park, PA, United States

**Keywords:** code-switching, complementizers, equivalence constraint, prosodic variation, syntactic variation, processing cost

## Abstract

The contentious question of bilingual processing cost may be recast as a fresh question of *code-switching (CS) strategies—*quantitative preferences and structural adjustments for switching at particular junctures of two languages. CS strategies are established by considering prosodic and syntactic variables, capitalizing here on bidirectional multi-word CS, spontaneously produced by members of a bilingual community in northern New Mexico who regularly use both languages (Torres Cacoullos and Travis, 2018). CS strategies become apparent by extending the equivalence constraint, which states that bilinguals avoid CS at points of word placement conflict (Poplack, 1980), to examine points of inconsistent equivalence between the languages, where syntactic difficulty could arise. Such sites of *variable equivalence* are junctures where the word strings of the two languages are equivalent only sometimes due to language-internal variable structures. A case in point for the English-Spanish language pair is the boundary between main and complement clauses, where a conjunction occurs always in Spanish but variably in English. The *prosodic distancing strategy* is to separate the juncture of the two languages. Here the complement clause appears in a different prosodic unit from the main clause—disproportionately as compared both with monolingual benchmarks and with bilinguals’ own unilingual English and Spanish. Prosodic distancing serves to mitigate variable equivalence. The *syntactic selection strategy* is to opt for the variant that is more quantitatively available and more discourse neutral. Here the preference is for the Spanish complementizer *que—*regardless of main or complement clause language. This is the more frequent option in bilinguals’ combined experience in both their languages, whereas the English complementizer *that* is subject to a number of conditioning factors. Syntactic selection serves to restore equivalence. Discovery of community CS strategies may spur reconsideration of processing cost as a matter of relative difficulty, which will depend on bilinguals’ prosodic and syntactic choices at particular CS sites.

## Introduction

Code-switching (CS) may be defined as stringing together two languages in alternation. In (1), for example, the speaker begins the sentence in Spanish, continues in English, and ends in Spanish (In the examples, stretches of speech originally produced in English are italicized in the translation on the right.) CS is generally agreed to be orderly, though debate continues over the rules governing it (Poplack, 2015: 918). The notion that CS incurs blanket processing cost, however, is contentious (see Johns et al., 2019: 585–587 for a review). In this article, the question of cost is refashioned into an investigation of bilingual CS strategies. We establish prosodic distancing and syntactic selection strategies, capitalizing on CS data by members of a bilingual speech community who regularly use both languages. CS strategies are discoverable in speakers’ structural choices, as revealed by distribution patterns in the spontaneous production of CS.


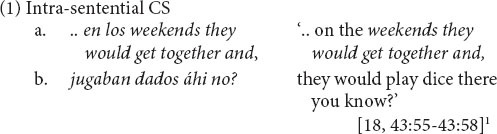


### Modulation of CS Cost

Though psycholinguistic studies resting on a range of behavioral and brain imaging measures widely report CS costs, the extent and even applicability of CS cost is controversial. One issue is that cost may pertain to cued rather than natural production, as when participants are required to name items (for a review of the language switching experimental paradigm see, e.g., Litcofsky and Van Hell, 2017: 113). Yet more generally, findings of bilingual processing costs are increasingly acknowledged to be contingent on study participants, experimental design and language mixing type.

First, as concerns participants, a crucial factor is linguistic experience with CS, which modulates presumed cognitive costs. Studies with university student participant pools tend to privilege relative language proficiency as assessed via formal tests and questionnaires (rather than language use as observed via a sociolinguistically constructed corpus). Yet cognitive-neurological consequences of bilingualism, for example, involving executive control, are likely affected by what has been called “the behavioral ecology of bilingual speakers” (Green, 2011: 1) or “participants’ code-switching habits” (Hofweber et al., 2016: 648). In particular, processing costs may “depend on the frequency of code-switching in the bilingual community” (Adamou and Shen, 2019: 53). Because “the behavior of an individual can be understood only through the study of the social groups of which he or she is a member” (Labov, 2010: 7), the question of bilingual linguistic experience thus becomes one of discovering speech community norms for CS.

Second, reported CS costs depend on experimental design. The tasks asked of participants, but also the stimuli and the way they are presented, turn out to be pivotal. For example, an eye-tracking study reported greater processing difficulties when participants were asked for an acceptability judgment on the code-switched sentence they had read than when they were asked a comprehension question about the sentence’s content (Guzzardo Tamargo, 2012; Beatty-Martínez et al., 2018: 9). In a word recognition task, reaction times were found to be similar with verbs in mixed sentences from other languages as with the verbs in unilingual sentences, for members of a community where other-language verb insertions occur regularly in everyday speech; in contrast, “ecologically non-valid” stimuli yielded slower reaction times (Adamou and Shen, 2019: 66). At the same time, even for “valid” stimuli, the manner of presentation affects the outcome. For example, a mixed mode with unilingual and code-switched sentences resulted in shorter processing times than a blocked mode with an all-unilingual block and an all-code-switches block. This result would be consistent with natural production, in which “intra-sentential code-mixing does not occur for long stretches of time and is broken up by unilingual discourse” (Johns et al., 2019: 584).

Third, for language mixing type, a key consideration is the extent of the material from the other language. Most neurocognitive studies reporting switching costs have been restricted to single-word other-language items (as noted by, e.g., Litcofsky and Van Hell, 2017: 113–115), giving short shrift to multi-word string combinations or CS of the “alternational” type (Muysken, 2015: 259).

In sum, despite an abundance of lab-based studies, assessing CS cost is far from a settled matter. Here we shift perspectives, recasting the question of cost as one of CS strategies to contribute findings from actual performance, relying on a well-defined bilingual community, the data of everyday speech and a uniquely large sample of multi-word CS. It has been proposed that CS serves as a general “strategy for optimizing task performance” (Beatty-Martínez et al., 2020). We propose to identify particular CS strategies, by considering the role of prosodic and syntactic variables.

### CS Strategies

CS cost may be viewed as a matter of degree. Such an approach parallels psycholinguistic findings for degrees of processing difficulty in monolingual language use. For example, English object relative clauses (*They were good herring that we got*) are less frequent than subject relatives (*It’s your arteries that fur up*), on a scale of approximately 3 to 1 in everyday speech (Tagliamonte et al., 2005: 87). Object relatives are also more difficult to process. But the difficulty is modulated by both online contextual features (such as animacy of the head noun) and cumulative linguistic experience with object relatives (statistical learning) (e.g., Wells et al., 2009: 87; Hsiao and MacDonald, 2016: 250). Following from a view of difficulty as a relative concept, instead of assuming blanket CS cost as compared with monolingual processing, CS may be more difficult at some junctures of the two languages than at others.

CS strategies are seen in the preferences for CS at particular sites and the ways of dealing with those CS sites that are not preferred. We thus define *CS strategies* as quantitative preferences and structural adjustments for CS at particular junctures of the two languages. CS theories have been mostly concerned with constraints on CS, that is, permissible CS sites. From the perspective of CS strategies, however, the twin questions are the following: (1) Of the permissible sites, are there ones bilinguals actually prefer? (cf. Sankoff and Poplack, 1981: 37) and (2) How do bilinguals treat prosodic and syntactic boundaries at the junctures of their two languages?

## Method and Materials

### Intra-Sentential CS and Prosodic Structure

In order to identify CS strategies, we focus on multi-word strings from two languages within an integrated sentence, or intra-sentential CS (cf. Poplack, 1980: 589). This is because syntactic difficulty should be minimal for alternating entire sentences, for example, in response to a change in topic or interlocutor.

How are sentences delimited in speech? The quality of the transcription of speech corpora is often a serious drawback. A well-established replicable method is based on the Intonation Unit (*IU*), a segment of speech produced with “a single, coherent intonation contour” (Du Bois et al., 1993: 47; see Appendix 3 for acoustic features)^2^. In the example of spontaneously produced CS in (1) above, each line of transcription represents an IU. Punctuation at the end of each line represents types of transitional continuity between IUs. A comma indicates “continuing” intonation, projecting more to come, as in (1a) and (2a), (2b) below, while a period marks “final” intonation—a fall to low pitch—as in (2c), and a question mark, “appeal” —a high rise in pitch—as in (1b) (Du Bois et al., 1993: 52–55). See Appendix 1 for transcription conventions.

Intra-sentential CS is operationalized for spoken discourse on the basis of the “prosodic sentence,” illustrated in (2): one or more clauses in one or more IUs, the last of which ends in intonational completion, represented by a period or question mark (Chafe, 1994:139–140). Inter-sentential CS may be inter- or intra-clausal (Deuchar, 2020: 2). Within the prosodic sentence in (2), the first switch in line (a) is inter-clausal (at the juncture of two adverbial clauses), while the CS between lines (b) and (c) is intra-clausal (at the juncture of adverb and negated finite verb within a single clause).


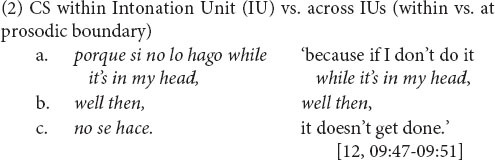


Prosodically based transcription is particularly handy for identifying CS patterns. In (2), note that the first instance of CS occurs within the IU [in line (a)] and the second at IU boundaries [between lines (b) and (c)]. We now know that CS is more frequent at the boundary of prosodic units (captured here across lines) than within them (Torres Cacoullos and Travis, 2018: 51–52; cf., Mettouchi, 2008: 195; Shenk, 2006: 189; Steuck, 2018). Intra-sentential multi-word CS in the NMSEB corpus occurs at a rate that is approximately three times greater across IUs than within IUs (in aggregate across different syntactic boundaries, where the universe is the total number of IUs eligible to host CS) (Torres Cacoullos and Travis, 2020).

### Equivalence Constraint

Over the years, many CS theories have appealed to some notion of equivalence or congruence between languages (e.g., Deuchar, 2005: 255; Lipski, 1978: 257–258; Muysken, 2015: 259). Of the many available theories of CS, the equivalence constraint (Poplack, 1980: 58l) is readily operationalizable into predictions that can be tested in a corpus of bilingual speech. In addition, it neither assumes that bilingual patterns need be derivable from syntactic principles for monolingual grammar nor depends on theory-internal postulates and thus may facilitate comparisons across studies.

The equivalence constraint states that CS tends to occur at syntactic boundaries present in both languages, or conversely, CS is avoided at points of word placement incompatibility between the two languages (Poplack, 1980: 586; Sankoff, 1998: 46–47). Proposed and operationalized in Poplack’s (1980: 590–595) community-based study of spontaneously produced CS, this simple constraint accounted for nearly all occurrences. Fewer than 1% (*n* = 11/1,835) of switches occurred at points where the word orders of the two languages were different (Poplack, 1980: 611). Also argued to be consistent with equivalence as a condition on CS are findings for cross-language syntactic priming, which is favored when word order is homologous across languages (e.g., Loebell and Bock, 2003: 227; Kootstra et al., 2010: 808).

To illustrate for English and Spanish, let us take adjectives as a site of CS (3). Attributive adjectives tend to occur post-nominally in Spanish but are prenominal in English. CS between attributive adjective and noun is restricted (largely to a small set of prenominal Spanish adjectives), whereas there is a propensity to switch before a predicative adjective*—*a point at which the languages are compatible (Sankoff and Poplack, 1981: 33). Even among equivalence sites, though, there may be preferences. For example, the boundary between verb and lexical object shows a somewhat higher CS rate than that between lexical subject and verb (Sankoff and Poplack, 1981: 35; cf. Poplack, 1980: 604).


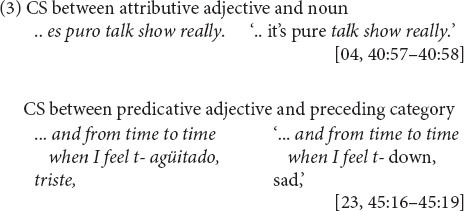


### Variable Equivalence

CS strategies may be most clearly observed where syntactic difficulty could arise, at points of inconsistent compatibility, or sites of *variable equivalence*. These are points where the word strings of the two languages are equivalent only sometimes, at syntactic boundaries that occur variably in one or both of the languages (Torres Cacoullos and Poplack, 2016). How do bilinguals deal with CS at sites of variable equivalence? The answers will allow us to discern structural adjustments for CS at particular junctures of the two languages.

The juncture between main and complement clause, illustrated in (4) and (5), is a site of variable equivalence for English-Spanish bilinguals. This is because of the inconsistent compatibility between English and Spanish in the presence of the complementizer. In English, complementizer *that* is present only sometimes. Rates of complementizer *that* presence range approximately between just 10% and 30% in corpora of spoken English (e.g., 9%, *n* = 4,106, Tagliamonte and Smith, 2005: 299; 21%, *n* = 2,820, Torres Cacoullos and Walker, 2009: 20; 34%, *n* = 3,681, Wulff et al., 2018: 105). Complementizer absence, as in (6), is thus the majority variant. (In the examples, absence of *that* is indicated with a Ø between the main clause [MC] and the complement clause [CC]^3^.)

In Spanish, in contrast, the complementizer *que* is present, as in (7), virtually always (Silva-Corvalán, 1994: 137). An exception is particular well-wishing formulaic expressions (as with first person, present-tense *espero* ‘I hope’) (Rodríguez Ricelli, 2018: 323–327). Thus, due to language-internal structural variability, complementizer presence is not an equally probable choice across the two languages. The differing probabilities of the analogous options (*that*, *que*) within each language make the main and complement clause boundary a site of variable equivalence for CS between the languages.


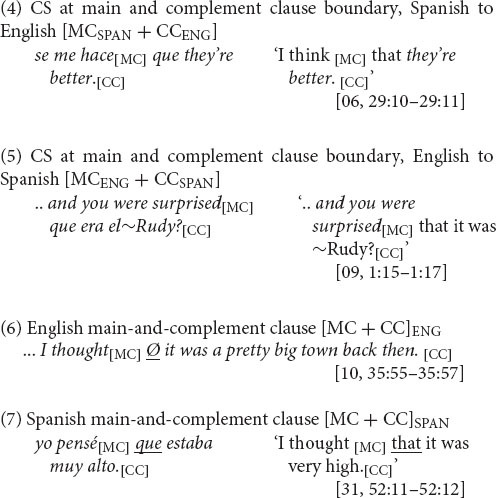


### CS Between Main and Complement Clauses in the Literature

The main topic sparked by CS between main and complement clauses has been the language of the complementizer, in other words, whether the switch is *after* the complementizer, remaining in the language of the main clause, or *at* the complementizer, initiating the switch to the language of the complement clause.

Proposals have swung according to the prevailing formal syntactic theory (see Pérez-Leroux et al., 2014: 284–285, 291 for a summary), on the assumption that bilingual, CS-particular rules are unwarranted (e.g., Vergara, 2018: 234 and references therein). For example, the complementizer has been argued to be in the language of the main clause verb, under the notion of a “government” relation between sentence constituents (Di Sciullo et al., 1986). The contrary claim has also been made, that switching is banned between the complementizer and the subordinate clause, based on the notion of a “functional head” (Belazi et al., 1994). Under a newer Chomskyan notion of “phase,” the prohibition against switching between complementizer and complement clause would hold for certain (“plain featureless”) complementizers (e.g., “that” vs. “since”) (López et al., 2017: 9–10).

Violations of such principles have been handled by a model for bilingual phenomena in which complementizers come from the Matrix Language (ML). Thus, with Spanish-English CS, both possibilities are allowed as long as the ML is identified accordingly: [MC_SPAN_ + *que* + CC_ENG_] and [MC_ENG_ + *that* + CC_SPAN_], where the ML is that of CP1, as well as [MC_SPAN_ + *that* + CC_ENG_] and [MC_ENG_ + *que* + CC_SPAN_], where the ML is that of CP2 (Myers-Scotton and Jake, 2009: 352; CP = Complementizer Phrase). Taken to support a ML account, for example, has been Igbo-English CS, where the complementizers are in the language of the (Igbo) main clause (Ihemere, 2016: 177–178), but also literary Spanish-English CS, where a “majority” of complementizers are in the language of the complement clause (Callahan, 2004: 50).

Another topic has been the appearance of the Spanish complementizer *que* in an otherwise unilingual English sentence, as in *I always got the feeling, que he was never comfortable.* [15, 38:04–38:07]. Lone other-language complementizers may be borrowed (Matras, 2009: 287; Joseph, 2016: 196) or on a “continuum from borrowing to CS” (Treffers-Daller, 2005: 500), an instance of “leaks” (Bentahila and Davies, 1998: 42) or of “congruent lexicalization” (Muysken, 2015: 244–247). Socially, such items may be ethnic identity markers (Pfaff, 1979: 314, referencing Gumperz and Hernández-Chávez, 1975: 156–157 on interjections and connectors). Lone complementizer *que* must be dealt with elsewhere, here appearing sparsely (*n* = 7 tokens vs. *n* = 467 unilingual English sentences in which it could have appeared, produced by 5 of 40 speakers (see Data)).

This embarrassment of proposals brings us to the question of data sources and test criteria. In the following, rather than selected counterexamples to categorical constraints, we look to quantitative patterns and speaker preferences.

### CS Strategies: Prosodic Distancing and Syntactic Selection

We entertain the following hypotheses about bilingual strategies at sites of variable equivalence:

#### Prosodic Distancing Strategy

Mitigate variable equivalence by prosodically separating the juncture of two languages.

#### Syntactic Selection Strategy

Construct consistent equivalence by opting for the more readily available syntactic variant.

According to the prosodic hypothesis, bilinguals use prosody to distance CS boundaries at sites of variable equivalence. This is based on the generalization that there is a tighter syntactic relationship between words in the same Intonation Unit (IU) than between words positioned in different IUs (Croft, 1995: 849–864). For example, while main clauses tend to appear in separate IUs from one another, as in (1), complement clauses tend to be prosodically integrated in the same IU with their main clauses (Croft, 1995: 861). This is true for both English and Spanish, that is, main and complement verbs tend to occur in the same IU, as in (6) and (7) above^4^. The prosodic hypothesis predicts that the prosodic integration of main and complement clauses will diminish when CS occurs between them.

The syntactic hypothesis states that bilinguals create consistent equivalence for CS at sites of variable equivalence. How? In the case of variable complementizer presence, they would restore equivalence by using a complementizer, eschewing the complementizer absence option. Now, whether complementizers remain in the language of the main clause or initiate the switch into the language of the subordinate clause (see preceding section), in switching to or from Spanish, bilinguals would use English *that* at a higher rate than in monolingual English main-and-complement clause structures. Conversely, they may prefer the Spanish complementizer *que*, regardless of CS direction. If so, the prediction is for a predominance of [MC_ENG_ + *que* + CC_SPAN_] and [MC_SPAN_ + *que* + CC_ENG_], as in (4) and (5) above, over [MC_ENG_ + *that* + CC_SPAN_] and [MC_SPAN_ + *that* + CC_ENG_].

To verify bilingual strategies, the procedure is to extract all tokens of CS at a particular site and compare their behavior with those of unilingual and monolingual counterparts at the CS-hosting site. Let us first contextualize the data.

### Community and Corpus

The New Mexico Spanish-English Bilingual (NMSEB) corpus consists of 31 recordings (2010–2011) with 40 members of a long-standing, non-immigrant, bilingual community, all speakers who regularly use both their languages (Torres Cacoullos and Travis, 2018: 13–73). Spanish and English have coexisted as the main competing languages for over 150 years in the northern region of New Mexico, a United States southwestern state (Bills and Vigil, 2008: 29–47). The speakers of the NMSEB corpus are Hispanic New Mexicans, 23 women and 17 men, born between 1923 and 1989. They include mineworkers, ranchers, and a variety of service employes, and most (29/40) live in rural areas.

The participants are members of a *speech community*, a group of individuals sharing “well-defined [geographic] limits, a common structural base and a unified set of sociolinguistic norms” (Labov, 2007: 347). As an established *bilingual* speech community, they also share unified conventions for combining their languages (Torres Cacoullos and Travis, 2018: 25). As an example of bilingual community norms for combining languages, consider the preferred way to incorporate English-origin verbs into Spanish. This is with light verb *hacer* ‘do,’ e.g., *lo hic-ieron* [do-PFV.3PL] *hire* ‘they hired him,’ in New Mexican, but not in Puerto Rican, Spanish (cf. Wilson and Dumont, 2015: 450–451).

Community norms, furthermore, may impact the neurology of language control (Green, 2011: 2). One dimension is degree of bilingualism. CS cost has been associated with language dominance and thus imbalance in switching direction—from L1 to L2 vs. L2 to L1 (e.g., Pérez-Leroux et al., 2014: 303–307). Dominance in turn has been inferred from self-rating scales, language history questionnaires, standardized proficiency tests, and online measures such as from picture naming or verbal tasks (for a review of the construct, see Treffers-Daller, 2016). Alternatively, dominance may be viewed through frequency and domains of use of two languages (Treffers-Daller, 2019: 385–388). For the NMSEB corpus, the scores for, and lack of correlation between, language preference, self-rating, and predominance (proportion of clauses produced in each language) give no ground for designating either English or Spanish as the dominant language (Appendix 2). The participants are highly bilingual, as validated in the aggregate by the stretches of English and Spanish in even amounts (Torres Cacoullos and Travis, 2018: 57–73).

Moreover, seamless CS corroborates the speakers’ bilingualism. Northern New Mexico bilinguals may change languages with no particular rhetorical or interactional motivation (Torres Cacoullos and Travis, 2018: 67–71). In such “intra-situational” CS, the two languages are brought together in a single speech event, with no change in interlocutor or topic, that is, no external trigger (Poplack, 2015: 918). For these bilinguals, CS functions as an “appropriate” (Gonzales, 1999: 29) “overall discourse mode” (Poplack, 1980: 614). They would thus seem to be prime candidates for exemplifying what some call “habitual codeswitchers” (e.g., Fricke et al., 2016: 111). It has been proposed that, as these code-switchers do not “avoid” switching, “their skill lies less in avoiding language conflict than in utilizing the joint activation of both languages” (Green, 2011: 2).

Finally, having defined the participants, we record non-elicited CS. The most systematic production data for linguistic analysis are provided by the vernacular—the unreflecting use of language when minimum attention is paid to monitoring speech (Labov, 1972: 112). For speakers of stigmatized varieties, especially, experimental procedures may evoke educational institutions, where the speakers and their local varieties have been denigrated, eliciting formal self-monitored responses (Sankoff, 1988: 145). Thus, for the NMSEB corpus spontaneous speech data were recorded by community in-group members through sociolinguistic interviews (Labov, 1984: 32–42; Travis and Torres Cacoullos, 2013: 178–181).

### Data

The data consist of finite verbs with finite clausal complements. For English, excluded are collocations such as *I think* and *I guess* occurring alone in their own IU, which may function as epistemic adverbials rather than main verbs (Thompson, 2002: 142; Torres Cacoullos and Walker, 2009: 9; Travis and Torres Cacoullos, 2014: 364–365). For Spanish, not counted as a complement-taking verb is *es que* ‘it’s that’ (as in .. *es que I teach them a lot no?* ‘it’s that *I teach them a lot no?*’ [20, 1:09:49]). See Steuck (2016: 77–80) for data extraction protocols.

The CS dataset is the subset of main-and-complement clause complexes in which the change in language occurs at the clause boundary, as in (4) and (5), which, as discussed earlier, is a site of variable equivalence. These switches are distinguished from intra-clausal instances in which CS occurs within the main or complement clause but not at the boundary between them, as in (8).


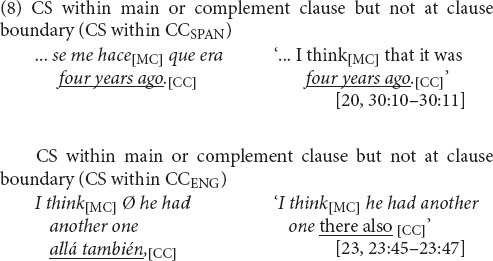


Also counted separately are cases of single-word items, mostly lone English nouns as in (9), which tend to be syntactically integrated into Spanish as the recipient language (Torres Cacoullos and Aaron, 2003: 466; Aaron, 2015; cf. Sankoff et al., 1990; Poplack, 2018).


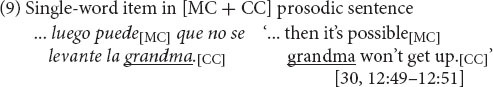


Excluded are cases in which CS occurs following final intonation, that is, outside the target prosodic sentence, as in (10), where the complete syntactic unit in line (a) is extended with an “increment” in line (b) (cf. Ford et al., 2002: 16).


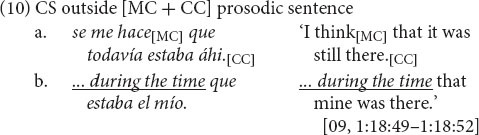


**FIGURE 1:**
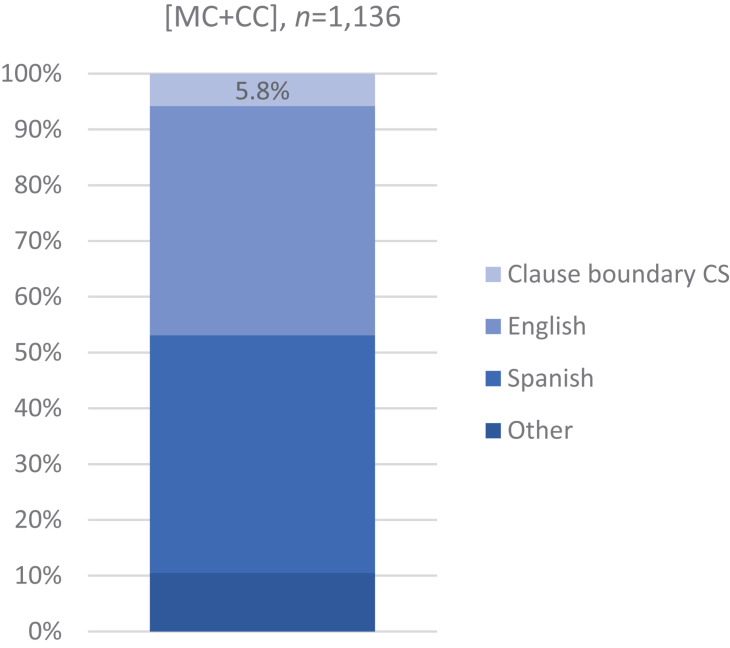
CS rate: Bilinguals’ main-and-complement clause sentences according to language (*n* = 1,136). Most main-and-complement clause sentences are unilingual, divided between English (41%, *n* = 467) and Spanish (43%, *n* = 484). The rate of CS at the main and complement clause juncture is 5.8% (*n* = 66). Other: [MC + CC] with CS other than at clause boundary or with single-word items.

As seen in Figure 1, most of the main-and-complement clause sentences are unilingual, fairly evenly split between English (41%, *n* = 467) and Spanish (43%, *n* = 484). CS at the main and complement clause boundary occurs at a rate of 5.8% (*n* = 66). The remaining cases are of intra-clausal multi-word CS at other than the clause boundary [as in (8)] (*n* = 44) and single-word items (*n* = 75). The CS dataset is, to our knowledge, the largest [66 tokens of switching at the complement clause boundary, versus, for example, a total of 76 relative and subordinate clauses of all kinds (Poplack, 1980: 602)].

To verify bilingual strategies, we will be comparing CS tokens with their unilingual as well as monolingual main and complement clause counterparts. Monolingual benchmarks are comparable speech corpora prosodically transcribed following the same protocols, the Santa Barbara Corpus of Spoken American English (Du Bois et al., 2000–2005) and the Corpus of Conversational Colombian Spanish (cf. Travis, 2005: 9–25).

## Results

### Prosodic Strategy: Distance the Language Boundaries

As introduced above, the prosodic CS strategy states that bilinguals tend to prosodically distance a variably equivalent juncture of the two languages. The prediction here is based on what we know about monolingual main and complement clauses, which cross-linguistically tend to occur in the same IU (Croft, 1995: 861). Rates of realization of complement-taking verbs and their complement in one IU have been reported to be 78% (*n* = 844) in English and 68% (*n* = 328) in Spanish conversational data (Steuck, 2016: 81). Following the prosodic distancing hypothesis, we may thus predict that when CS occurs at the boundary between main and complement clauses, they will be integrated in the same IU at a lower rate than their unilingual and monolingual counterparts.

**FIGURE 2:**
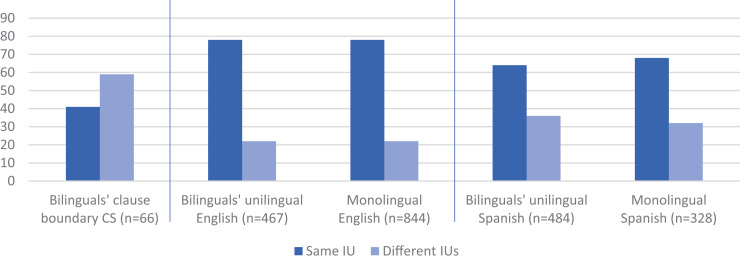
Prosodic realization of main-and-complement clause [MC + CC]:The tendency to occur in the same IU obtains in bilinguals’ unilingual sentences (English 78%, *n* = 467; Spanish 64%, *n* = 484), at rates closely matching their respective monolingual benchmarks (English 78%, *n* = 844; Spanish 68%, *n* = 328), but not when CS occurs at the main-and-complement clause boundary (41%, 27/66) (From Steuck and Torres Cacoullos, 2019: 223, 227).

Precisely such is the result, seen in Figure 2. The tendency is for integration in the same IU in bilinguals’ unilingual sentences (English 78%, *n* = 467; Spanish 64%, *n* = 484), at rates closely matching their respective monolingual benchmarks (Steuck and Torres Cacoullos, 2019: 223, 227; a Fisher’s exact test comparing bilinguals’ unilingual English with monolingual English showed no difference in integration rates, *p* = 1.00 and, similarly, no difference between bilinguals’ Spanish and monolingual Spanish, *p* = 0.291; the difference in integration rates between monolingual English and Spanish, *p* = 0.0005, is maintained between bilinguals’ English and Spanish, *p* < 0.0001).

But with CS between main and complement clause the tendency is the *opposite*, to occur in different IUs, as in (11) and (12) (with a rate of realization in one IU of just 41%, 27/66) (see Appendix 3). This prosodic separation strategy holds in both switching directions. For English to Spanish (11), the main and complement clauses appear in different IUs at a rate of 58% (18/31) and for Spanish to English (12), 60% (21/35).


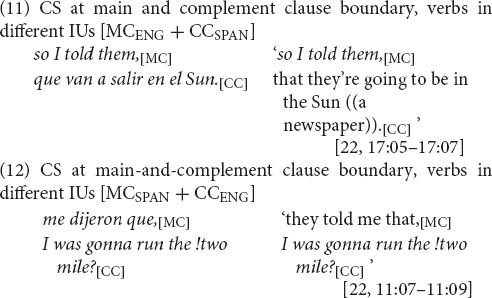


It is important to bear in mind that the prosodic distancing of main and complement clauses when CS occurs at their juncture is not a mere reflection of the general preference to switch across rather than within IUs (which holds across intra-sentential CS sites in aggregate, see section “Intra-sentential CS and Prosodic Structure”). When CS is intra-clausal, that is, when CS occurs as part of the main or complement clause—but not at their juncture—as in (13) (and [8] above), the tendency for prosodic integration stands (with a rate of realization of main and complement verbs in the same IU of 64%, *n* = 44) (Steuck and Torres Cacoullos, 2019: 227). This set of findings—that main-and-complement clause sentences with CS *other than* at the clause juncture are realized prosodically the same way as bilinguals’ unilingual main-and-complement clause sentences, which in turn align with their monolingual benchmark counterparts—is evidence that prosodic distancing is not due to some intrinsic cost of CS. Rather, prosodic distancing responds to variable equivalence, serving to mitigate the inconsistent compatibility at this particular juncture.


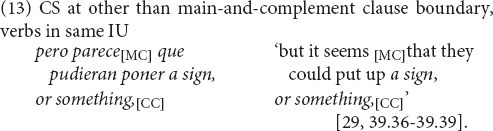


### Syntactic Strategy: Select an Equivalence-Restoring Variant

**FIGURE 3:**
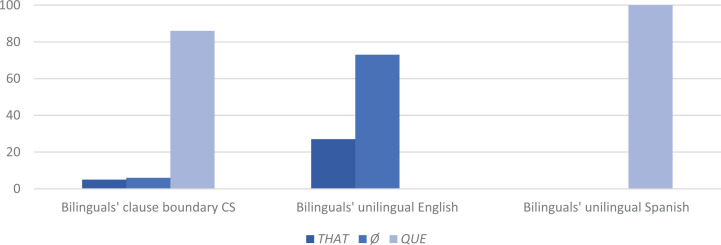
Use of complementizers according to main-and-complement clause language (*n* = 1,017): Bilinguals’ unilingual complementizer use adheres to the respective patterns of each of their languages (in unilingual English, complementizer that is variably present, at a rate of 27% (126/467); in unilingual Spanish, complementizer que is 100% present). With CS at the clause boundary, complementizer *que* predominates, at 86% (57/66).

We hypothesized that a syntactic strategy for variably equivalent junctures in a language pair is to opt for the more readily available variant from one of the languages, so that the syntactic boundary between languages is realized as a site of equivalence. For the main-and-complement clause boundary, this is tested by the presence of the complementizer when CS occurs. Before considering those results, a prior result, depicted in Figure 3 (middle and right columns), is that bilinguals’ unilingual complementizer use adheres to the respective patterns of each of their languages. In bilinguals’ unilingual English main-and-complement clause complexes, complementizer *that* is variably present, at a rate of 27% (126/467), within the range reported for monolingual English (see section “Variable Equivalence” above). In their unilingual Spanish, complementizer *que* is always present, as in monolingual Spanish. We confirm, thus, that complementizer presence is indeed a site of variable equivalence for these speakers.

Furthermore, besides adhering to monolingual rates, bilinguals’ English maintains the constraints on complementizer use. English complementizer *that* presence is subject to lexical, discourse and structural factors (see, e.g., Shank et al., 2016: 202–213; Wulff et al., 2018: 100–101 and references therein). The conditioning factors indicate that English *that* is used to demarcate clauses when both have semantic or propositional content (Torres Cacoullos and Walker, 2009: 29). Material intervening between the main clause verb and the complement clause favors the presence of *that*, as do lexical rather than pronominal subjects in the complement clause. Particular main-clause verbs, especially first-person subject and simple present-tense forms such as *I think*, *I guess*, *I remember*, in contrast, are associated with absence of *that*. Bilinguals’ English shows parallels with this linguistic conditioning of variable *that* presence, which is more frequent when there is intervening material than when there is not (46%, 52/112 vs. 21%, 74/355) and with other than first-person singular main clause subjects (58%, 65/113 vs. 17%, 16/353).

The remarkable result is that, despite the integrity of complementizer use in bilinguals’ unilingual English and Spanish as separate languages, at their juncture there is strong skewing in favor of one of the options, as depicted in Figure 3 (left set of columns). Given the nearly even numbers of English and Spanish unilingual main-and-complement clause complexes, we might have expected that with CS at the clause boundary, the distribution of complementizer options would be proportional: roughly 10% *that*, 40% Ø (complementizer absence), and 50% *que.* Instead, complementizer *que* predominates, at 86% (57/66). The remaining cases are 4 of complementizer absence, 3 of *that*, and 2 of *that que*.

An important aspect of this result is that of all instances of CS at the main and complement clause juncture, only 6% are with the Ø option—complementizer absence—as in (14) (compare (15)). The explanation is that the main and complement clause boundary does not occur in the absence of a complementizer in Spanish. The disproportionate preference for complementizer presence in CS, then, constitutes a reconstruction of equivalence between English and Spanish in word string patterns.


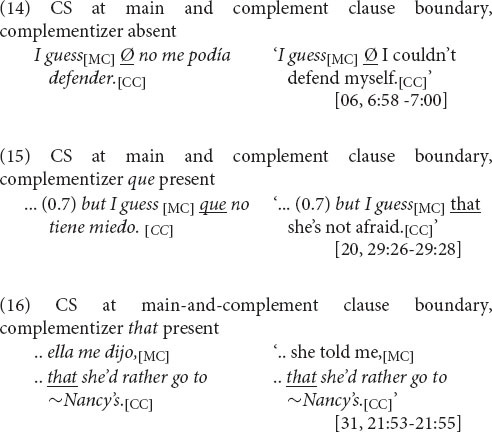


**FIGURE 4:**
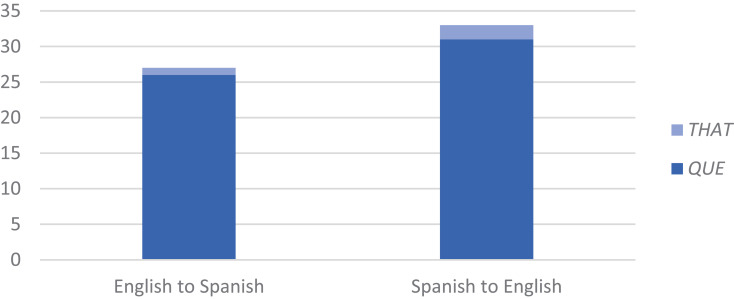
Use of complementizer according to CS direction (n = 60): Spanish *que* predominates over English *that*, regardless of CS direction and the language of the main verb, at a rate of 96% (26/27) when CS is from English to Spanish and 94% (31/33) when CS is from Spanish to English. CS goes from English to Spanish and Spanish to English in about even proportions (47%, 31/66 and 53%, 35/66).

The other important aspect of this result is that, of the 94% of CS instances with the complementizer present, nearly all are with Spanish *que*, while instances with English *that*, as in (16), constitute only 5% (3/66). This is a genuine preference, not an accident of data distributions. As seen in Figure 4, CS goes from Spanish to English and English to Spanish in about even proportions (53%, 35/66 and 47%, 31/66). That is, in nearly half the instances of CS at the clause boundary, the direction is English to Spanish, and still Spanish *que* predominates over English *that*, at a rate of 96% (26/27). This is virtually identical to the rate of *que* when CS goes in the opposite direction, from Spanish to English, at 94% (31/33). (The number of observations in Figure 4 is 60, excluding cases of complementizer absence (*n* = 4) and of *that que* (*n* = 2).) A compatible result has been reported from an elicited oral production task, where subjects produced *que* more often than *that* whether the stimulus began in Spanish or English (Dussias, 2001: 33).

**FIGURE 5:**
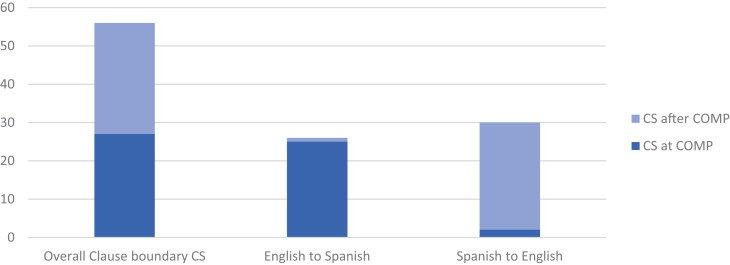
Position of CS at main-and-complement clause boundary—beginning at complementizer versus after complementizer—overall and according to CS direction (*n* = 56): The positioning of CS overall is approximately even, occurring 52% (29/56) after, 48% at, the complementizer; English to Spanish CS tends to occur at the complementizer, 96% (25/26), Spanish to English CS after the complementizer, 93% (28/30).


Figure 5 now shows the positioning of the switch. This may be *after* the complementizer, such that main verb and complementizer are in the same language, as in (17), and *at* the complementizer, such that complementizer and subordinate verb are in the same language, as in (18). The positioning of CS is approximately even, occurring 52% (29/56) after, and 48% at the complementizer (left column in Figure 5). (The number of observations in Figure 5 is 56, not counting cases with an intervening clause between main and complement clauses (*n* = 4), for example, *oh yeah I remember, .. cuando llegaba gente, que nos decía*, ‘*oh yeah I remember*, .. when people would visit, that she would tell us,’ [03, 37:04]; counting these, the proportion of CS occurring after the complementizer is 53% (32/60).)

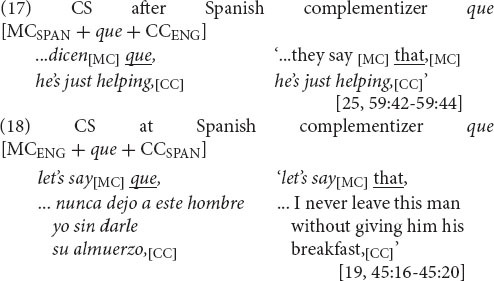


As also indicated in Figure 5, the positioning of the switch, at or after the complementizer, and the direction of the switch, are highly correlated: English to Spanish CS tends to occur at the complementizer, 96% (25/26), Spanish to English CS tends to occur after the complementizer, 93% (28/30). These patterns contradict constraints derived from (monolingual) syntactic theories and supported by introspective or elicited judgments, anecdotal observations or haphazardly collected examples. The generalization can be neither that “the complementizer [.] is in the same language as the main verb” (Di Sciullo et al., 1986: 8) nor that “the complementizer is in the language of the complement clause” (Belazi et al., 1994: 224). Rather, this correlation follows from bilinguals’ strong preference to use *que* for CS at the clause boundary—regardless of the language of either the main or the complement clause.

In sum, bilinguals do not overuse minor English option *that*, but select major Spanish option *que*. They prefer the Spanish complementizer regardless of CS direction. As predicted in accordance with the syntactic selection strategy for variable equivalence, [MC_SPAN_ + *que* + CC_ENG_] (*n* = 28) and [MC_ENG_ + *que* + CC_SPAN_] (*n* = 25) predominate over [MC_SPAN_ + *that* + CC_ENG_] (*n* = 2) and [MC_ENG_ + *that* + CC_SPAN_] (*n* = 1). With the structural adjustment of syntactic selection—preferential selection of a syntactic option from one of the languages—the syntactic boundary becomes one that occurs in both languages, and equivalence is restored.

## Discussion

Bilingual CS strategies are quantitative preferences and structural adjustments for switching at particular junctures of the two languages. Adopting a view of processing cost not as inherent to CS but as a relative concept, we zeroed in on particular structural junctures of the two languages: sites of variable equivalence, where word placement is not always realized in the same way in both languages. Such is the main-and-complement clause boundary in English and Spanish, where the complementizer is variably present in one language but categorically present in the other. Though, theoretically, bilinguals could resolve variable equivalence through grammatical convergence (e.g., by “dropping” Spanish *que* on the model of English *that* or by extending *that* on the model of *que*), in this bilingual community the conflict between the two languages remains intact (cf. Poplack and Levey, 2010).

The prosodic distancing strategy is to separate the boundary between languages, here via the appearance of the complement clause in a different prosodic unit from the main clause, disproportionately as compared with unilingual and monolingual benchmarks. Prosodic distancing mitigates the problem of variable equivalence by disconnecting the juncture of the languages where a word placement conflict may arise. The syntactic selection strategy is to recruit the more available option from one of the languages at the boundary with the other, here Spanish complementizer *que*. Choosing such a syntactic option bypasses the problem of variable equivalence, by reconstructing equivalence.

How to explain the disproportionate preference for the Spanish complementizer? One explanation would posit that Spanish is the Matrix Language (ML) providing the morpho-syntactic frame in main clauses, such that “a strong preference for the ML to supply ‘that-type’ complementizers at clause boundaries is predicted”; in other words, “whatever ML dominates in the discourse seems to preference complementizers from that language” (Myers-Scotton and Jake, 2009: 355)^5^. The prediction is not upheld half the time, no matter how the ML is assigned (Figure 5). The positioning of CS—at versus after the complementizer—is approximately even, such that the complementizer is in the same language as the complement clause as often as it is in the same language as the main verb.

Beyond the particular prediction, inconsistent with CS models assuming that one language dominates is, for one, that the distribution of main-and-complement clause sentences by language is about even between English and Spanish (Figure 1), which renders inconsequential such a posited asymmetry between the two languages within the corpus. Remember, too, that there is no overextension of *that*, unlike Spanish-speaking L2 learners (Wulff et al., 2018: 118): bilinguals’ English maintains monolingual English patterns for prosodic integration and for complementizer *that* rate (Figure 2, Figure 3). Furthermore, the bidirectionality of the multi-word CS indicates that these bilinguals have real choices, not imposed by language dominance (Figure 4; cf. Appendix 2). In sum, the distribution of clauses by language, patterns of language-internal variability, and directionality of CS in the northern New Mexico corpus would not justify conferring on one of the languages the status of an overarching matrix language.

Rather, the preference for the Spanish complementizer is a genuinely bilingual strategy, for which both languages come into play. Bilinguals opt for Spanish complementizer *que* to construct equivalence because it is the more available variant, according to a usage-based approach to linguistic structure and process (e.g., Bybee, 2010). How so? For one, *que* is the *quantitatively* more readily available option. In bilinguals’ cumulative linguistic experience, given the use of both their languages, *que* will be more frequent than *that*. This status of *que* as the major variant thanks to its greater frequency would not be inconsonant with an explanation that has been suggested for choosing *que* over *that* on the basis of a weaker bond between verb and complementizer in English than in Spanish (Dussias, 2002: 34).

Spanish *que* is also what we might call the more *neutral* option. English complementizer *that* has grammaticalized from its origins as a demonstrative pronoun and its diachronic trajectory may be one of increasing use, but it remains variable (Shank et al., 2016: 237; cf. Hopper and Traugott, 1993: 185–189). Variable *that* use is subject to discourse contextual factors such as the form-topicality of the complement clause subject, whereas Spanish *que* is an obligatory marker of a clause as a complement (Thompson and Mulac, 1991: 248; Torres Cacoullos et al., 2017, 79–81). The linguistic conditioning of *that* makes it a more meaningful—and less neutral—variant than *que*. The more context-independent Spanish complementizer is thus a more likely candidate than the probabilistically constrained *that* for constructing equivalence at the juncture of the two languages.

The **syntactic selection strategy** for CS may therefore be more precisely restated as follows:

Construct consistent equivalence by opting for the quantitatively more available and more discourse-neutral variant from one of the languages.

The implications for lab-based experiments on CS cost would follow from the dictum that “production predicts comprehension” (Hsiao and MacDonald, 2016: 87; cf. Johns et al., 2019: 599 and references therein). For English-Spanish bilinguals, we can predict that CS at the main-and-complement clause boundary will be more difficult to process relative to Intra-sentential CS elsewhere, for example, when the switch point is before a predicate adjective, an adverbial expression or a lexical direct object (see examples (3), (8), and (13)). Cost should be attenuated with distancing of the clause boundary by placing the clauses in separate prosodic units or, in written stimuli, perhaps through use of commas or appearance on separate lines. And we expect cost to be reduced in the presence of Spanish complementizer *que* compared with English *that*. Lab-based work could also investigate combinations of syntactic and prosodic options, for example, less-preferred *that* together with separation of the clauses.

More general hypotheses may be stated as follows. First, CS will be more difficult to process—by appropriate behavioral or neurophysiological responses (and with caveats concerning experimental design and participants’ bilingual practices)—at some boundaries than at others. Difficulty is modulated by variable word placement conflicts at the junctures of two languages. And second, at sites of variable equivalence CS will be less difficult when (1) the juncture of the two languages is prosodically distanced and (2) a frequent and neutral equivalence-restoring syntactic variant is selected, according to bilingual community conventions.

These findings emerge from the spontaneous language-combining behavior of bilinguals for whom using both languages and alternating between them is an everyday occurrence. It is bilinguals’ choices that enable us to discern CS strategies.

## Data Availability Statement

The datasets for this manuscript are not publicly available to protect participants against misunderstanding of local variants and the unintentional publication of stereotyping examples by those unfamiliar with the speech community. Unlike much of the data generated in lab-based bilingualism studies, sociolinguistic corpora are re-usable and citable. Such data are a more accurate representation of actual performance, sometimes of a highly personal nature in interactions with in-group interviewers, and often from members of close-knit minority language communities. In accordance with the ethical commitment to the speakers not to place the data in the public domain, individual requests for access may be directed to the author.

## Author Contributions

RTC contributed to the manuscript with 100%.

## Conflict of Interest

The author declares that the research was conducted in the absence of any commercial or financial relationships that could be construed as a potential conflict of interest.

## Footnotes

2 For the NMSEB transcriptions (see Community and Corpus), each hour of recorded speech required minimally 50 h, in five rounds, by three trained transcribers (Torres Cacoullos and Travis, 2018: 47–51).

3 In the examples, subscripts identifying main [MC] and complement clause [CC] are positioned such that the complementizer is associated with the [CC], but no statement is intended as to whether the complementizer belongs with the main or complement clause.

4 Prosodic separation of main and complement clauses is probabilistically favored by intervening material and also main clause subjects other than first person (Steuck, 2016: 88), that is, predicates with more semantic substance (cf. Ono and Thompson, 1995: 238–242).

5 Thanks to a reviewer for pointing out the predicted preference for main-clause ML complementizers (Myers-Scotton and Jake, 2009: 355) countered here, as well as the statement, not contradicted here, that “*que* can introduce an English CP [.] and *that* [.] a Spanish CP” (2009: 352) (see Section 2.4).

## Appendix 1

Transcription Conventions (see Du Bois et al., 1993)^∗^.

**Table:**

Carriage return New Intonation Unit (IU) (where the IU does not fit on one line, the second line is indented)			

.	final intonation contour	..	short pause (0.2 secs)
,	continuing intonation contour	…	medium pause (0.3–0.6 secs)
?	appeal intonation contour	…()	timed pause (0.7 secs or longer)
∼	pseudonymized proper noun	(())	researcher’s comment
!word	speech pronounced with notably high pitch		

^∗^Symbols for vocal noises, laughter, and lengthening have been removed for the purposes of readability.

## Appendix 2

New Mexico bilingual community members according to language preference, self-rating, and predominance, all speakers (*n* = 40) and, in second columns, those producing CS at main-and-complement clause boundary (*n* = 19).

**Table:**

	Both		English		Spanish	
Reported preferred language	8	6	20	7	12	6
Relative self-rating	25	14	8	3	7	2
Predominance in use*	15	10	13	4	12	5

^∗^ Speakers were categorized as “English predominant” if more than two-thirds of their total clause count (finite verbs) were English and “Spanish predominant” if more than two-thirds were Spanish, or else “Both” (total *n* clauses = 36,000), as a measure of relative level of use and activation. Preferred language and self-rating scores are not strongly correlated; language predominance does not correlate with reported language preference nor strongly with self-rating (on the pitfalls of transposing proficiency assessments into the community setting, see Torres Cacoullos and Travis, 2018: 58–72).

## Appendix 3

Occurrence in a single Intonation Unit and in separate IUs of main and complement clauses with CS at the clause boundary, after and at the complementizer (examples (4) and (18)).^∗^

^∗^ Delimiting IU boundaries is done perceptually (Ford and Thompson, 1996: 145–149). Features used by the transcriber include higher pitch at the beginning of the IU and a slower speech rate at the end (as with *better* in the first example), and pausing between IUs (in the second example).
